# GAIT UNSTEADINESS AS AN INDICATOR OF COGNITIVE STATUS IN INDIVIDUALS WITH PERIPHERAL NEUROPATHY

**DOI:** 10.1093/geroni/igz038.3110

**Published:** 2019-11-08

**Authors:** Gu Eon Kang, Jacqueline Yang, He Zhou, Changhong Wang, Bareera Akhtar, Brian xu, Guillermo Beckmann, Bijan Najafi

**Affiliations:** Baylor College of Medicine, Houston, Texas, United States

## Abstract

Neuropathic individuals are at risk of falls, however potential impact of cognitive impairment in neuropathic individuals is not well-understood. Since cognitive impairment is considered an independent risk factor for falls, knowing its potential, additional impact may help better understand underlying mechanism of risk of falls in neuropathic individuals. We aimed to investigate stride-to-stride variability in neuropathic individuals with cognitive impairment (NP-Cog-Impaired) and without cognitive impairment (NP-Cog-Intact) during normal and dual-task walking. Neuropathic symptoms and cognitive status was measured using maximum vibration perception threshold (VPTmax) in the feet and the Montreal Cognitive Assessment (MoCA), respectively. We analyzed data from 19 NP-Cog-Impaired (8 men; 68.5±9.1 years; 29.0±6.2 kg/m2; VPTmax=27.2±12.1 volts; MoCA=19.6±2.4) and 25 NP-Cog-Intact (15 men; 66.5±9.1 years; 31.3±5.9 kg/m2; VPTmax=26.3±12.7 volts; MoCA=25.6±1.6). We collected movement data using five inertial sensors (LEGSysTM, BioSensics LLC, Watertown, MA) attached on the shanks, thighs and lower back. We used previously validated algorithm to calculate coefficient of variations (CV) of stride velocity and stride length. CV of stride velocity and stride length were significantly greater for the NP-Cog-Impaired group (11.07±5.22% and 7.31±3.20%, respectively) than for the NP-Cog-Intact group (7.31±3.20% and 4.81±2.80%, respectively) for dual-task walking but not for normal walking. Between normal and dual-task walking, CV of stride velocity and stride length increased 43.2% (significantly) and 46.4% (marginally), respectively, from normal walking to dual-task walking for the NP-Cog-Impaired group but not for the NP-Cog-Intact group. Results suggest that cognitive impairment may be an additional risk factor of falls in neuropathic individuals.

